# MicroRNA-223-3p is involved in fracture healing by regulating fibroblast growth factor receptor 2

**DOI:** 10.1080/21655979.2021.2002498

**Published:** 2021-12-07

**Authors:** Bin Wang, Wei Wu, Ke Xu, Haihao Wu

**Affiliations:** Orthopaedic Centre, Hwa Mei Hospital, University of Chinese Academy of Sciences, Ningbo, Zhejiang, China

**Keywords:** Mir-223-3p, fracture healing, fgfr2

## Abstract

MicroRNAs (miRNAs) are powerful modulators of fracture healing. The research explored the level of serum miR-223-3p in fracture patients and its potential mechanism in fracture healing. In the study, miR-223-3p levels in 42 patients with intra-articular fracture and 40 patients with hand fracture were detected by real-time fluorescence quantitative PCR reaction (qRT-PCR). Subsequently, osteoblasts MC3T3-E1 was transfected with miR-223-3p mimic or inhibitor, and cell function was detected by Cell counting kit (CCK-8) assay and flow cytometry. Dual-luciferase reporter assay verified the regulation mechanism of miR-223-3p and its target genes. We found that miR-223-3p was significantly elevated over time in patients with intra-articular fracture and hand fracture compared with healthy individuals. Moreover, increased miR-223-3p significantly reduced cell viability and promoted cell apoptosis. The fibroblast growth factor receptor 2 (FGFR2) was the target of miR-223-3p. Serum FGFR2 was significantly decreased in patients, which was contrary to the expression of miR-223-3p. Moreover, FGFR2 levels in cells were negatively regulated by miR-223-3p. Finally, si-FGFR2 significantly reversed the promotion of miR-223-3p inhibitor on cell viability and the inhibition of cell apoptosis. Our research suggested that miR-223-3p is highly expressed in fracture patients, and regulates osteoblast cell viability and apoptosis by targeting FGFR2. This may be a valuable target for fracture healing therapy and provide a new perspective for its treatment.

## Introduction

Fracture is a common injury that can occur at all ages and is often caused by impact, stress, or disease [1]. It an estimated that more than 16 million fractures occur in the United States each year [2]. Although fractures have a certain healing ability, 5%-10% of fracture patients still have delayed healing or non-healing, leading to limited walking, chronic pain, which seriously damages the quality of life of patients and puts a huge burden on families and society [3]. Fracture healing is a complicated and slow process of repair [4]. It involves systemic or local circumstances, together with some types of cells and growth factors that communicate with adjacent tissues and blood to promote bone healing [5]. The current treatment strategy for nonunion of fractures requires a second operation and cannot guarantee the success rate of fracture union [6]. Therefore, it is necessary to have an in-depth understanding of the fracture healing process and related mechanisms, and appropriate intervention is helpful to accelerate bone regeneration.

As small single-stranded RNA molecules, microRNAs (miRNAs) participate in the disease progress through the negative regulation of target genes. In the field of bone biology, miRNA has been proven to regulate the differentiation of osteoblasts and become a key regulator of bone formation, resorption, repair, healing, and even bone-related diseases [7]. Such as exosome miR-128-3p targets SMAD family member 5 (SMAD5) to promote fracture healing [8]. miR-497-5p is involved in the role of long non-coding RNA PVT1 in promoting fracture healing [9]. MiR-25-5p promotes osteoblast differentiation by down-regulating SMAD7 to inhibit runt-related transcriptional factor 2 (RUNX2) expression under hypoxia [10]. Importantly, Waki et al. analyzed the miRNA profile of rat fracture nonunion and found 5 highly expressed miRNAs, including miR-223-3p [7]. Deep vein thrombosis, a common complication of fracture, is also regulated by miR-223-3p [11]. Besides, studies have reported that miR-223-3p regulates the differentiation of osteoblasts by regulating the feedback loop [12]. Although the role of miR-223-3p in these studies has been described in detail, its role in fracture healing is not yet fully understood.

Based on the above studies, we speculated that miR-223-3p has a special role in fracture healing. Therefore, we discussed this speculation and its possible mechanisms, to find a new therapeutic strategy for fracture healing.

## Materials and Methods

### Collection of serum samples

This study was approved by the Medical Ethics Committee of Hwa Mei Hospital, University of Chinese Academy of Sciences (Ningbo No. 2 Hospital), and was carried out with the informed consent of the subjects. According to the protocol approved by the declaration of Helsinki, all samples have been obtained and anonymized.

A total of 82 patients with fractures who were treated at Hwa Mei Hospital, University of Chinese Academy of Sciences (Ningbo No. 2 Hospital) from January 2016 to December 2018 were included. Inclusion criteria for patients included: (1) patients with skeletal maturity, (age ≥ 18 years); (2) fresh fracture (days < 3 weeks); (3) patients with low-energy post-traumatic fragility fracture or on trauma after trauma. Patients with autoimmune diseases, infected wounds, or severe amputations were excluded. The 82 patients included had 42 intra-articular fractures (24 males, 18 females; mean age 49.14 ± 16.24 years; mean BMI: 26.45 ± 1.75 kg/m^2^) and 40 hand fractures (29 males, 11 females; mean age 50.70 ± 13.69 years; mean BMI: 26.00 ± 1.57 kg/m^2^). 70 healthy individuals (41 males, 29 females; mean age 46.39 ± 13.62 years; mean BMI:25.99 ± 1.54 kg/m^2^) were selected from the healthy examination center as the control group, and patients with joint, chronic inflammation, and autoimmune deficiency were excluded. Blood samples were collected on the day of admission and days 7, 14, and 21 after standardized fixed treatment. After standing, the upper serum was collected by centrifugation and stored at −80°C. In addition, basic clinical information of the subjects included in the study is shown in Table 1.

**Table 1.** Comparison of the baseline data of study objects

Parameters	Healthy individuals (n = 70)	Intra-articular fracture (n = 42)	Hand fracture (n = 40)	*P value*
Gender (female/male)	29/41	18/24	11/29	0.268
Age (year)	46.39 ± 13.62	49.14 ± 16.24	50.70 ± 13.69	0.291
BMI (kg/m^2^)	25.99 ± 1.54	26.45 ± 1.75	26.00 ± 1.57	0.292

Annotation: BMI, body mass index. Data are presented as mean ± standard deviation.

### Cell culture and Transfection

Osteoblast MC3T3-E1 was obtained from BeNa Culture Collection. The cell culture was supplemented with α-modified minimal essential medium (α-MEM) in which 10% FBS and 1% mixture of penicillin and streptomycin were added.

MC3T3-E1 (passage number 7–12) was inoculated into a culture dish and transfected with a fusion rate of 70%. Transfection vectors were miR-223-3p mimic (sequence: 5ʹ-UGUCAGUUUGUCAAAUACCCA-3ʹ), miR-223-3p inhibitor (sequence: 5ʹ-UGGGGUAUUUGACAAACUGACA-3ʹ), negative control miR-NC (sequence: 5ʹ-UUUGUACUACACAAAAGUACUG-3ʹ), si-FGFR2 (sequence: 5ʹ-AGCCCUGUUUGAUAGAGUAUATT-3ʹ), and si-NC (sequence: 5ʹ-UUCUCCGAACGUGUCACGUTT-3ʹ), respectively. The transfection reagent was Lipofectamine 2000. Specific transfection process: mix the transfection reagent with transfection reagent, incubate at room temperature, and then drop the mixed solution into the 6-well plates. After 6 h in the incubator, replace with fresh medium.

### RAN extraction, reversal, and real-time fluorescence quantitative PCR (qRT-PCR) reaction

TRIzol reagent was added to serum and cells to isolate and extract total RNA. After the quality of the extracted RNA was identified with a spectrophotometer (RNA purity was determined by the A260/280 ratio range of 1.8–2.1), the total RNA extracted was reverse-transcripted into cDNA with miRcute Plus First-Strand cDNA Kit. Then, using 2 muL cDNA as template, and use miRcute Plus miRNA qPCR Kit (SYBR Green) to perform qRT-PCR amplification on the MX3000p Real-time PCR apparatus. Amplification was 10 min of initial setup at 95°C, followed by 40 amplification cycles (15 s of denaturation at 95°C and 60s of annealing/extension at 60°C). Ce-miR-39 and β-actin were used as internal reference genes. The primers were as follows: miR-223-3p forward: 5ʹ-UGUCAGUUUGUCAAAUACCCCA-3ʹ, reverse: 5ʹ-CAGTGCAGGGTCCGAGGT-3ʹ; ce-miR-39 forward: 5ʹ-TCACCGGGUGUAAATCAGCTTG-3ʹ, reverse: 5ʹ-CAGTGCAGGGTCCGAGGT-3ʹ; FGFR2 forward: 5ʹ-TGACATTAACCGTGTTCCTGAG-3ʹ, reverse: 5ʹ-TGGCGAGTCCAAAGTCTGCTAT-3ʹ; β-actin forward: 5ʹ;-TCACCCACACTGTGCCCATCTACGA-3ʹ and 5ʹ-CAGCGGAACCGCTCATTGCCAATGG-3ʹ. The expression levels of miRNA and FGFR2 were calculated by 2^−ΔΔCt^, where CT is cycle threshold, and ΔCt = CT (miRNA or FGFR2)-CT (reference genes).

### Cell viability assay

Cell viability was measured by a CCK-8 assay [13]. The transfected cells were inoculated into 96-well plates at a concentration of 2 × 10^3^ cells. The original medium was removed at 0, 24, 48, 72 h, respectively, and DMEM 100 μl containing CCK-8 reagent was supplemented. The cells were incubated in the incubator for 1 h and then vibrated on a microtiter plate reader for 30 s. The changes in the OD value at 450 nm were detected. Cell viability was calculated according to OD values of 3 days t.

### Apoptosis assay

Apoptosis ability was analyzed by Annexin V-fluorescein isothiocyanate (FITC)/propidium iodide (PI) kit (4A Biotech, Beijing, China) and flow cytometry to determine the cell distribution at four different quadrants, namely viable cells, early apoptosis, late apoptosis, and necrotic cells. The transfected cells were digested with EDTA-free trypsin solution and collected with PBS. Then resuspended with 200 μl Binding buffer. Annexin V-FITC 10 μl and 5 μl PI were supplemented and incubated for 5 min in the dark. Finally, the number of apoptotic cells was detected by flow cytometry. The total percentage of apoptotic cells was defined as the sum of both early apoptosis (annexin V-FITC positive, PI negative) and late apoptosis (annexin V-FITC PI-positive) [14].

### Dual-luciferase reporter assay

A fragment of FGFR2 containing either the wild-type (WT, sequence: 5ʹ-CCUGUUAAUUUUUAUACUGACAA-3ʹ) or mutant (MUT, sequence: 5ʹ-CCUGUUAAUUUUUAUUGACACAA-3ʹ) miR-223-3p binding sites was then synthesized by Sangon Biotech, and then inserted into the luciferase reporter pmiRGLO vector. MC3T3-E1 cells were inoculated into 24-well plates, and after overnight culture, vectors containing luciferase reporter gene and miR-223-3p mimic or inhibitor were mixed with transfection reagents in vitro. After standing at room temperature for 20 min, it was dripped into cells. The luciferase activity was detected 48 h after transfection [15].

### Statistical analysis

The GraphPad Prism 6.0 software was applied to statistical analysis. Student t-test was used to determine statistical significance between two groups, while a one-way ANOVA, followed by Tukey’s multiple comparison post-hoc test, was used to determine statistical significance between three or more groups. All experiments in this research had at least 3 independent biological replicates. *P* < 0.05 was believed a statistically significant difference.

## Results

42 intra-articular fractures, 40 hand fractures, and 70 healthy controls were included in the study. The expression levels of miR-223-3p and its potential target in the serum of the subjects were analyzed. In addition, we attempted to regulate the levels of miR-223-3p and target genes in osteoblasts to explore its effects on cell proliferation and apoptosis in fracture healing. To confirm the hypothesis that miR-223-3p may participate in fracture healing by regulating its potential target genes.

### Serum miR-223-3p was upregulated in fracture patients

**Figure 1.**
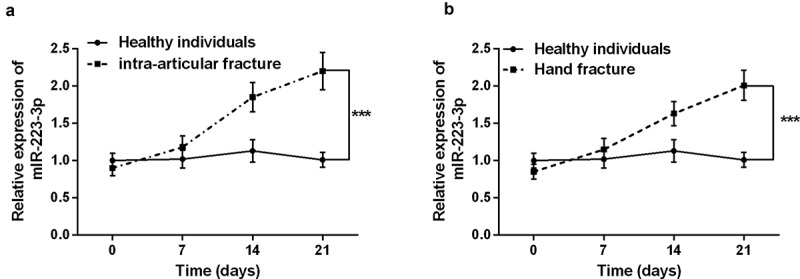
Serum levels of miR-223-3p in patients with fractures were detected by the qRT-PCR reaction. a. The level of miR-223-3p in intra-articular fracture patients increased gradually over time. b. The level of serum miR-223-3p in hand fracture patients gradually increased over time. *** *P* < 0.001, compared with healthy individual

The basic information of the subjects was analyzed first, and the results confirmed that there was no significant difference in gender, age, and BMI among the three groups (*P* > 0.05, Table 1). Subsequently, serum miR-223-3p in patients with intra-articular fractures and hand fractures was discovered by qRT-PCR. It can be seen from Figure 1a that serum miR-223-3p level decreased slightly at the beginning of intra-articular fracture healing compared to healthy individuals. But over time, the patient’s serum miR-223-3p increased significantly (*P* < 0.001). Surprisingly, the same results were observed in the healing process of patients with hand fractures. The results indicate that dysregulation of miR-223-3p may be involved in the fracture healing process.

### miR-223-3p significantly inhibited cell viability and promoted cell apoptosis

**Figure 2.**
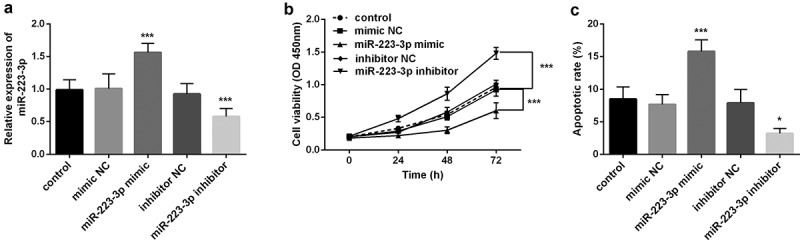
The level of miR-223-3p significantly regulated the cell viability and apoptosis of osteoblasts MC3T3-E1. a. Compared with miR-NC, miR-223-3p inhibitor decreased the level of miR-223-3p. b. cell viability was inhibited by overexpression miR-223-3p, while cell viability was promoted by miR-223-3p inhibitor. c. miR-223-3p mimic promoted cell apoptosis, while inhibition of miR-223-3p reduced cell apoptosis. *** *P* < 0.001, compared with miR-NC group

Furthermore, to investigate the influence of miR-223-3p on fracture healing, we transfected miR-223-3p mimic or inhibitor into osteoblast MC3T3-E1. The results showed that overexpression of miR-223-3p, the level of miR-223-3p was significantly increased, and miR-223-3p inhibitor significantly inhibited its level. The results confirmed that miR-223-3p could be regulated by mimic or inhibitor in vitro (*P* < 0.001, Figure 2a). Subsequently, the CCK-8 assay found that the increase of miR-223-3p level declined the cell viability of MC3T3-E1, while inhibition of miR-223-3p significantly greater the cell viability (*P* < 0.001, Figure 2b). Besides, in the apoptosis experiment, increasing miR-223-3p significantly promoted cell apoptosis, while decreasing miR-223-3p significantly inhibited cell apoptosis (*P* < 0.001, Figure 2c **and Supplementary data**). The results indicated that inhibition of miR-223-3p may promote fracture healing.

### MiR-223-3p directly targets FGFR2 in fractures healing

**Figure 3.**
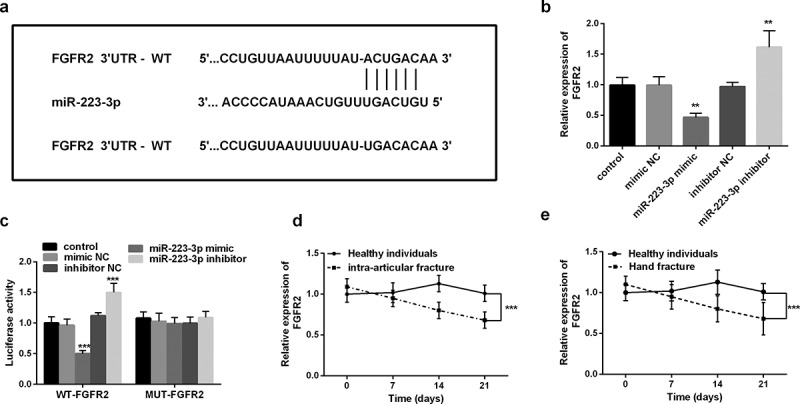
FGFR2 was the target of miR-223-3p. a. Schematic diagram of binging sites between FGFR2 mRNA 3ʹUTR sequence and miR-223-3p. b. FGFR2 mRNA levels can be regulated by different expression levels of miR-223-3p. c. Dual-luciferase reporter assay was used to determine the targeted binging of miR-223-3p and FGFR2. d. The levels of FGFR2 in intra-articular fracture patients. e. Levels of FGFR2 mRNA in patients with a hand fracture. ** *P* < 0.01, *** *P* < 0.001, compared with miR-NC groups

To understand the detailed mechanism of miR-223-3p regulating fracture healing, we studied the target of miR-223-3p in fracture healing. Bioinformatics software found that miR-223-3p had a binding site with the 3ʹUTR of FGFR2 (Figure 3a). Subsequently, we analyzed the changes in FGFR2 mRNA levels at different miR-223-3p levels. Increasing miR-223-3p significantly inhibited FGFR2 levels, while inhibiting miR-223-3p was the opposite (*P* < 0.01, Figure 3b). It is worth noting that the luciferase activity was markedly inhibited or increased after co-transfection of the mimic or inhibitor and WT FGFR2 3ʹUTR, while the luciferase actively of MUT FGFR2 3ʹUTR did not change (*P* < 0.001, Figure 3c).

Finally, we analyzed the changes in serum FGFR2 mRNA levels in patients with different fractures. As shown in Figure 3d-e, compared with healthy individuals, patients with intra-articular fracture and hand fracture had relatively higher FGFR2 levels at the beginning but decreased significantly over time (*P* < 0.001). These levels are in contrast to the pattern of serum miR-223-3p levels in patients. The above results all confirmed that miR-223-3p directly targets FGFR2 in fractures.

### miR-223-3p mediated osteoblast viability and apoptosis by targeting FGFR2

**Figure 4.**
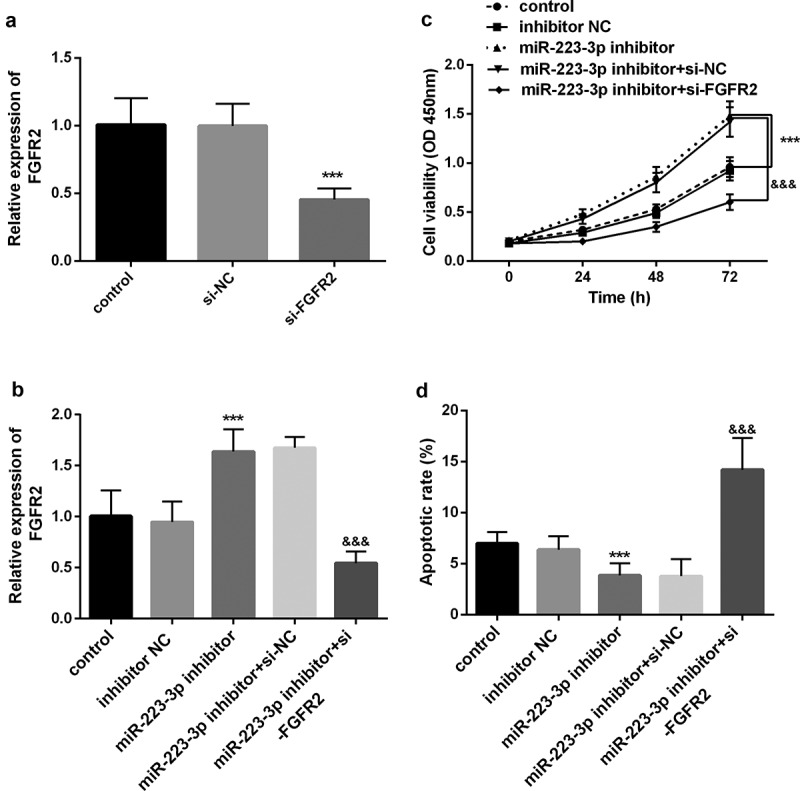
miR-223-3p was regulated cell viability and apoptosis by regulating FGFR2. a. si-FGFR2 markedly decreased the levels of FGFR2 in cells. b. si-FGFR2 reversed the promoting effect of the miR-223-3p inhibitor on the FGFR2 expression level. c. si-FGFR2 reversed the promotion of the miR-223-3p inhibitor on cell viability. d. si-FGFR2 reversed the inhibitory of miR-223-3p on apoptosis. *** *P* < 0.001, compared with miR-NC group. ^&&&^ *P* < 0.001, compared with miR-223-3p inhibitor+si-NC group

Previous results revealed that FGFR2 was the target of miR-223-3p. Subsequent research further proved that miR-223-3p requires FGFE2 to regulate the function of osteoblast MC3T3-E1. First, we knocked down FGFR2 in the cells and found that its level was markedly decreased (*P* < 0.001, Figure 4a). However, after being co-transfected with miR-223-3p inhibitor, the promotion effect of the miR-223-3p inhibitor on FGFR2 was significantly reversed (*P* < 0.05, Figure 4b). More importantly, si-FGFR2 significantly reversed the increase in cell viability and the decrease in apoptosis induced by a miR-223-3p inhibitor (*P* < 0.001, Figure 4c-d). The above results all confirm that miR-223-3p plays a key role in osteoblasts related to fracture healing by regulating FGFR2.

## Discussion

Fracture is common, and the pathologic and physiological mechanisms of fracture healing have not yet been elucidated to date. Fracture nonunion and delayed union of fractures bring serious economic and spiritual burdens to patients. However, there are currently no approved drugs to treat nonunion or accelerate bone healing, and there are no approved drugs to promote closed fracture healing [16]. Therefore, poor fracture healing remains a major clinical challenge. Current studies have proved that osteoblasts regulate new bone formation in fracture healing, and differentiate and proliferate under the influence of cytokines, hormones, growth factors, and other factors [17]. Although previous studies have confirmed that miR-497-5p [9] and others are involved in the process of fracture healing, miR-223-3p associated with osteoblasts was selected in this study.

MiR-223-3p is mainly located on the Xq12 chromosome, and its abnormal expression in a variety of diseases has been widely studied in recent years, such as gastric cancer [18], asthma [19], heart failure [20], acute kidney injury [21], diabetes [22]. miR-223-3p has been proven to be a powerful regulatory RNA in the field of bone biology. miR-223-3p is significantly elevated in patients with osteoarthritis and is involved in its pathogenesis [23]. Sinomenine regulation miR-223-3p overexpression and blocks IL-1β-induced chondrocyte apoptosis and protects osteoarthritis [24]. miR-223-3p is enhanced in mice with acute skeletal muscle injury [25]. Bone marrow mesenchymal stem cells (BMSCs) have the characteristics of self-renew and differentiate into osteocytes, chondrocytes, and adipocytes, which play a key role in bone formation. Studies have found that miR-223-3p regulates osteogenic differentiation of BMCS by targeting forkhead box O 1 (FOXO1) [26]. Furthermore, miR-223-3p is involved in the treatment of deep vein thrombosis, a common complication of fracture, by regulating angiogenesis and endothelial progenitor cell proliferation [11]. Osteoporosis is a common underlying cause of brittle fracture, and miR-223-3p has been reported as a disorder in patients with osteoporosis [27]. Importantly, Waki et al. analyzed the miRNA profile of fracture nonunion and found 5 highly upregulated miRNAs, including miR-223-3p [7].

Given the above findings, we speculate that miR-223-3p may participate in the regulation of fracture healing. To confirm our hypothesis, we recruited 42 patients with intra-articular fractures and 40 patients with hand fractures. We found that the serum miR-223-3p of both patients decreased slightly at the initial stage of fracture, but its levels increased significantly over time. This is consistent with miR-223-3p levels in other bone-related diseases. What’s more, fracture healing is a slow recovery process, mainly involved in the proliferation and apoptosis of osteoblasts, so regulating the activity of osteoblasts is helpful to fracture healing [28]. Osteoblasts are derived from BMSCs, and the regulation of BMSCs by miR-223-3p has been reported [26]. Therefore, we further study the functional regulation of miR-223-3p on osteoblasts. The results confirmed that the inhibition of miR-223-3p can significantly promote the viability of osteoblasts and inhibit cell apoptosis. While the increase of miR-223-3p had the opposite effect. The results confirmed that inhibition of miR-223-3p could promote fracture healing.

The fibroblast growth factor receptor (FGFR) family is a superfamily of immunoglobulins. Its structure consists of an intracellular tyrosine kinase domain and three extracellular immunoglobulin-like domains. This family contains different types of FGFR1-4 receptors, which bind to ligands and excitedly affect cell function [29]. Among them, FGFR2 has been found to play a key regulatory role in bone development. For example, FGFR2 is involved in the regulation of aqueous extract of Aralia echinocaulis Hand on fracture healing [30]. LIN00472 alleviates osteoporosis by upregulating FGFR2 expression by sponge miR-300 to regulate osteoblast differentiation [31]. Therefore, the targeting binding sequence of miR-223-3p and FGFR2 was discovered through bioinformatics software, and the increase of miR-223-3p levels can significantly inhibit the level of FGFR2. The dual-luciferase reporter assay also confirmed that FGFR2 is the direct target of miR-223-3p. In addition, in different patient studies, FGFR2 elevated at the beginning of fracture but decreased significantly over time. This is contrary to the expression level of miR-223-3p in patients. Finally, we transfected si-FGFR2 while inhibiting miR-223-3p, which reversed the inhibition of the decrease in cell viability and increased apoptosis caused by miR-223-3p.

However, the current research has several methodological limitations that need to be considered in future studies. Such as, ossification or mineralization is of certain importance to fracture healing. However, this study failed to timely focus on the effects of miRNA on mineralization and osteogenesis genes in fracture healing, which is a limitation of this study. In addition, researching cell culture severely limits the generality of this study, which is also the limitation of current research. And the protein level of FGFR2 and its specific mechanisms in fracture healing need to be supported by further studies.

## Conclusion

The study provides a new direction, theoretical basis, and target therapy for fracture healing. We confirmed that miR-223-3p is upregulated in patients with fractures. Moreover, miR-223-3p may regulate osteoblasts’ activity and apoptosis by targeting FGFR2. This provides a new perspective for the treatment of fracture healing.

## Supplementary Material

Supplemental Material

## Data Availability

Corresponding authors may provide data and materials.

## Highlights

1. miR-223-3p increased in a time-dependent manner in patients with fractures

2. Elevated miR-223-3p can reduce the cell viability of osteoblasts and promote cell apoptosis

3. FGFR2 is the target gene of miR-223-3p and is negatively regulated by it

4. Inhibition of FGFR2 reversed the down-regulation of miR-223-3p on cell viability and apoptosis.

## Declarations

**Ethics approval and consent to participate**

This study was conducted with the approval of the Medical Ethics Committee of Hwa Mei Hospital, University of Chinese Academy of Sciences (Ningbo No. 2 Hospital), and the participant’s informed consent. All samples were obtained and anonymized following the protocol approved by the declaration of Helsinki.
